# A cross-sectional study of exposure across social media platforms for the British Orthodontic Society retention awareness campaign: #HoldthatSmile

**DOI:** 10.1177/14653125211054859

**Published:** 2021-11-02

**Authors:** Yung Lam, Jadbinder Seehra, Stefan Abela, Martyn T Cobourne

**Affiliations:** Department of Orthodontics, Centre for Craniofacial Development & Regeneration, Faculty of Dentistry, Oral & Craniofacial Sciences, King’s College London, London, UK

**Keywords:** social media, tooth movement, retainers, orthodontics, patient, professional education

## Abstract

**Objective:**

The use of media campaigns in health promotion has become more common in recent years. #HoldthatSmile is a British Orthodontic Society life-long retention awareness campaign aimed at the general public and dental profession launched in 2017. This study investigated #HoldthatSmile exposure on social media platforms over a 12-month period following campaign launch.

**Design:**

A cross-sectional content analysis.

**Setting:**

Public-facing, English-language posts tagged #HoldthatSmile on the Facebook, Instagram and Twitter social media platforms.

**Methods:**

Data were collected relating to a 12-month period, from September 2017 immediately following campaign launch, to the end of September 2018. The primary outcome was exposure to the #HoldthatSmile campaign on the different platforms through analysis of posts, measurement of audience reached and engagement. Secondary outcomes included demographics of user-types and content analysis of reactive comments on posts.

**Results:**

A total of 205 relevant posts with #HoldthatSmile were identified on Twitter (n=90, 43.9%), Facebook (n=64, 31.2%) and Instagram (n=51, 24.9%) with an overall calculated audience reach of 108,807 individuals. There were 1849 reactions across the three platforms. The percentage of reactions that generated comments was low at 3%, 6.1% and 5.7% for Twitter, Facebook and Instagram, respectively. Just over three-quarters of users were either Dental Surgeries (53%) or Professional Dental Bodies (28%) and the vast majority were from the United Kingdom. Overall, most comments were positive (46%, n=36) or neutral (44%, n=35) with only 10% (n=8) negative. The overriding theme was a person’s name, with the user commonly typing a friend’s name in order to bring their attention to the post.

**Conclusion:**

There were a relatively low number of posts relevant to #HoldthatSmile on Twitter, Facebook and Instagram over the campaign’s first 12 months. However, the majority of these did convey positive or neutral messages.

## Introduction

Orthodontic retention is a subject as old as the specialty of orthodontics (Reitan, 1969; Rody Jr and Wheeler, 2017; Webster, 1948) with an evidence base demonstrating that the permanency of any orthodontic treatment can only be guaranteed through the strategy of long-term retention (Littlewood et al., 2016). It is important that patients understand this as part of the consent process before embarking on treatment and, importantly, after the completion of active treatment, to minimise the risk of relapse. However, there is evidence to suggest that this is often not the case (Lasance et al., 2020). A variety of long-term retention strategies are available for the patient and orthodontist to consider, but these essentially fall into one of two categories: long-term part-time wear of removable retainers or permanent fixed retention (Rody Jr and Wheeler, 2017).

The British Orthodontic Society (BOS) is the sole national representative body for orthodontists in the United Kingdom (UK) and a registered charity, promoting the study and practice of orthodontics through research and education, improving professional standards and educating the public about orthodontics and orthodontic treatment. As part of this remit, the BOS launched #HoldthatSmile in September 2017 (BOS, 2017a). This national awareness campaign was aimed at the general public and dental profession and highlighted the importance of wearing retainers as a means of reducing relapse after orthodontic treatment, while also promoting the need for life-long retention. The campaign was launched at the 2017 British Orthodontic Conference with a press conference and was fronted by a prominent UK orthodontist who is an authority on retention (Littlewood et al., 2016). The campaign consisted of a short cartoon animation aimed at younger patients (BOS, 2017b), a patient-focused video using layman terms (BOS, 2017c) and a video aimed at the dental profession highlighting current scientific evidence (BOS, 2017d). The campaign published targeted press releases to the national and dental press and used a number of social media platforms (SMPs) to promote the messages and distribute the videos, which included Facebook, Instagram, Twitter, YouTube, Google+ and LinkedIn, under the hashtag #HoldthatSmile.

The nature of SMPs makes them a useful tool for health promotion and awareness (Balatsoukas et al., 2015). The potential reach of a population regardless of age, socioeconomic status, demographics and ethnicity means SMPs can offer a cost-effective conduit for overcoming barriers commonly encountered with more traditional health promotion campaigns (Wakefield et al., 2010). A number of online campaigns have been very successful in promoting public health messages through the use of SMPs (Gosselin and Poitras, 2008; Gough et al., 2017; Lenoir et al., 2017; Livingston et al., 2013; Moreno et al., 2009; Thackeray et al., 2013; Zowawi et al., 2015) including the so-called Ice Bucket Challenge for the Amyotrophic Lateral Sclerosis Association, which ultimately involved more than 17 million people uploading videos watched by over 440 million individuals around the world (Koohy and Koohy, 2014; Sohn, 2017). However, while there are advantages for patients accessing and discussing health information online, there can also be drawbacks. SMPs are essentially unregulated and misleading information can be easily propagated, which can be detrimental for patients, have a potentially negative effect on patient-healthcare professional relationships and lead to feelings of anxiety and worry for patients who may access distressing information (De Martino et al., 2017; Smailhodzic et al., 2016). A significant proportion of orthodontic patients are teenagers and young adults for whom the use of SMPs is commonplace (Henzell et al., 2013). These platforms allow orthodontic patients to express and discuss their treatment experiences freely, in both positive and negative contexts (Graf et al., 2020; Noll et al., 2017; Rachel Henzell et al., 2014). Interestingly, among the orthodontic subject areas that have been investigated in relation to SMPs, orthodontic retention and retainer wear have been portrayed in a negative light on Twitter, primarily due to the effect of retainers on daily and social activities and their long-term maintenance (Al-Moghrabi et al., 2017). A national campaign to educate patients and dentists on the importance of retention was therefore timely.

The aim of this cross-sectional study was to investigate the exposure profile of #HoldthatSmile, through content analysis of posts identified on the SMPs Facebook, Instagram and Twitter.

## Materials and methods

This was a cross-sectional study investigating exposure of the BOS orthodontic retention awareness campaign #HoldthatSmile on the Facebook, Instagram and Twitter SMPs. Data were collected from all English-language public-facing posts containing the hashtag #HoldthatSmile relating to a 12-month period from September 2017 immediately following campaign launch to the end of September 2018. Information was collected on a single day for Instagram and Twitter, and over two consecutive days for Facebook. Exclusion criteria included non-English language posts and those not relevant to orthodontics, such as personal pictures with people smiling, advertisements and promotional posts just including the hashtag.

The primary outcome was exposure to the #HoldthatSmile campaign on the different SMPs through analysis of relevant posts, measurement of audience reached and engagement. Audience reach was defined as the number of individual users that would potentially be exposed to the #HoldthatSmile. In order to calculate this population and avoid duplication, the number of unique users (a single account that posted a relevant #HoldthatSmile post) was recorded and the sum of all their followers was used as the representative figure of audience reach. An audience reach metric was calculated for all relevant posts associated with each SMP. Audience engagement was measured by analysing different individual reactions to posts using the metrics of ‘likes’, ‘shares’ (or ‘retweets’ for Twitter) and ‘comments’ produced by #HoldthatSmile posts.

Secondary outcomes included the demographics of user-types posting content about the campaign and content analysis of reactive comments on posts. A scoping search was used to identify demographic categories for users. Six user groups were identified, which were classified into professional organisations and general public domains. The professional organisations were categorised as: (1) Professional Dental Bodies [PDB]; (2) Dental Surgeries [DS]; (3) Dental Professionals (dentists or orthodontists) [DP]; (4) Commercial Dental Companies [CDC]; while the public domains were categorised as: (5) Public–Individuals [PI] and (6) Public–Groups (including communities) [PG].

Content analysis incorporated transcripts of comments extracted verbatim to provide sentiment and themes. Themes were developed until they saturated cover of all the verbatim comments. Emoji images and symbols are now regularly used within digital communication to express emotions. Whilst most are obvious connotations of an expression, some can be ambiguous and left open to interpretation by the audience, particularly if without text content. For this study, interpretation was carried out by the first author (YL) with further interaction with a second author (MTC) if there was any ambiguity.

All data collected in this investigation were available in the public domain, therefore formal ethical approval was not required. This study involved the use of both qualitative and quantitative data.

## Results

A total of 279 posts were identified for the three SMPs over the observation period with 205 classified as relevant to the #HoldthatSmile campaign. Twitter had the highest number of relevant posts (n=90, 43.9%), followed by Facebook (n=64, 31.2%) and then Instagram (n=51, 24.9%). The overall calculated audience reach for these posts was n=108,807, with Facebook having the highest reach (n=64,710) followed by Twitter (n=28,019) and then Instagram (n=16,078). Table 1 shows the metrics of audience engagement with posts through reactions to #HoldthatSmile for each SMP. There were a total of 1849 reactions across the three SMPs. Interestingly, despite having the lowest number of posts and population reach, Instagram was associated with the highest level of audience engagement through posts. The action ‘like’ demonstrates positive support for post content and Instagram also had the highest proportion of ‘likes’, accounting for 94.3% (n=839) of engagement, followed by Facebook and Twitter with 67.8% (n=356) and 56.5% (n=245), respectively. However, no users shared any posts on the Instagram platform. Users reacting to a post with a comment is regarded as being the highest form of audience engagement and was also highest for Instagram. The percentage of reactions accounted for by comments were 6.1%, 5.7% and 3% for Facebook, Instagram and Twitter, respectively (Table 1). The results demonstrated Twitter to be the most effective at generating coverage for the campaign. Facebook was most effective at reaching the highest volume of users, yet Instagram was most effective at engaging users.

**Table 1.** Audience engagement with posts through reactions to #HoldthatSmile for Facebook, Instagram and Twitter.

	No. of posts	No. of unique users	Total No. of audience engagements	Likes (%)	Shares/retweet (%)	Comments (%)
Facebook	64	40	525	356 (68)	137 (26)	32 (6)
Instagram	51	30	890	839 (94)	0 (0)	51 (6)
Twitter	90	20	434	245 (56)	176 (40)	13 (4)

**Figure 1.**
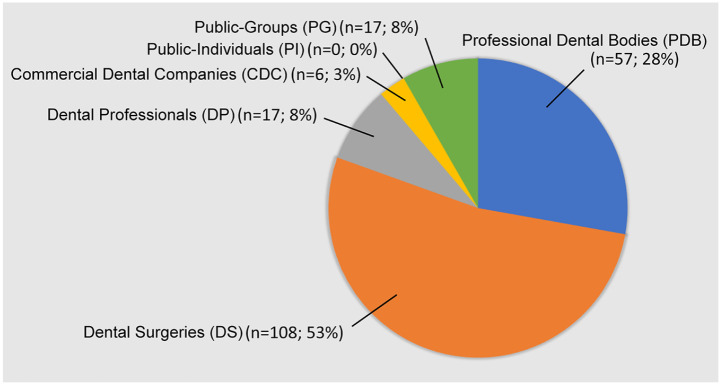
Distribution of user groups within the whole sample.

**Figure 2.**
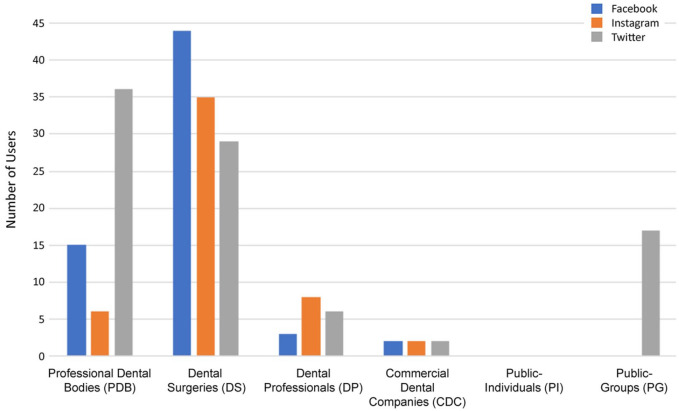
Distribution of user group for the Facebook, Instagram and Twitter social media platforms.

Figure 1 shows the distribution of user groups within the whole sample of 205. It can be seen that over three-quarters of users were designated as either DS (53%) or PDB (28%). The distribution of user group for each SMP is shown in Figure 2. The highest user group was DS for Facebook and Instagram, and PDB for Twitter. Interestingly, Twitter was the only SMP with users from the PG category and no users on any of the SMPs were designated as PI. Analysis of the geographical distribution of users showed that all platforms had a majority from the UK. Twitter had a 97% representation of UK user groups, then Instagram and Facebook with 94% and 89%, respectively. Facebook had the most diverse reach, with user group origins distributed among six further countries (Malta, Ireland, Singapore, Trinidad & Tobago, Canada and India) while Instagram (Malaysia, Lebanon, New Zealand) and Twitter (Pakistan, India, Saudi Arabia) both had three.

**Figure 3.**
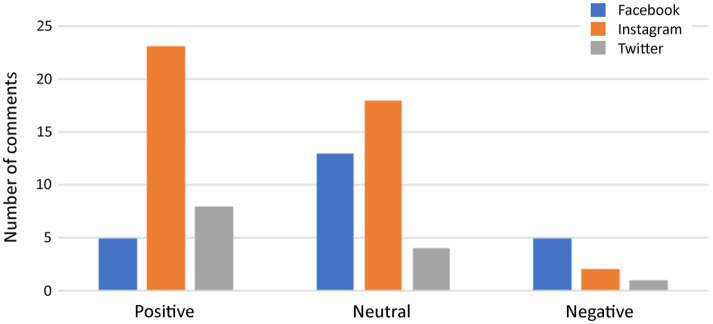
Comment sentiments for the Facebook, Instagram and Twitter social media platforms.

Content analysis of sentiments and themes was based upon 79 comments, which included all the Twitter comments (100%, n=13), 72% (n=23) of those from Facebook and 84% (n=43) from Instagram. Overall, the majority of comments were either positive (46%, n=36) or neutral (44%, n=35) with only 10% (n=8) being negative. Analysis of comment sentiments for the different SMPs revealed similar distribution patterns for Twitter and Instagram (Figure 3). The most common sentiments were positive for both these SMPs, with a distribution of 61% (n=8) and 53% (n=23), respectively. Neutral sentiments were represented by 31% (n= 4) for Twitter and 42% (n=18) for Instagram. Negative sentiments had the lowest numbers for both sites, with a contribution of 5% (n=2) and 8% (n=1), respectively. However, Facebook had an even distribution (22%, n=5) of positive and negative comments, and 56% (n=13) neutral comments. Eight themes arose from qualitative analysis of the content: (1) Person’s name; (2) Humour; (3) Dental health; (4) Retainers; (5) #HoldthatSmile campaign itself; (6) Positive or supportive comments about the campaign; (7) Negative comments about the campaign; and (8) Miscellaneous (advertisements, passing comments). The overriding theme was Person’s name (Table 2), with the user commonly typing a friend’s name in order to bring their attention to the post. This is a form of post-sharing but in a more specific targeted manner in raising awareness of the campaign. Humorous comments were also common and included smiling or crying emojis and comment such as, ‘I’m feeling the pain lol’ (where ‘lol’ is a means of expressing amusement and stands for ‘laugh out loud’). On Instagram, there was noteworthy positive support for the campaign with encouraging emojis such as thumbs-up and clapping symbols in common usage and comments such as ‘nice’ and ‘this is great’. However, nearly half the comments were not relevant to the campaign. There were also many users advertising themselves by enlisting links to their websites. Not all comments were available for content analysis because data could not be retrieved from the sites. Reasons for this included account users erasing comments, changing of account privacy settings preventing access or accounts no longer being active. Furthermore, after data collection it was apparent that replies are also included as comments by SMP automated calculations, but these were not analysed as part of the comments for this study. This contributed to a disparity between the number of comments analysed and the actual number recorded.

**Table 2.** Qualitative analysis of comment content for sentiments and themes.

	Facebook	Instagram	Twitter
Total No. of comments	32	51	13
No. of comments analysed	23 (72)	43 (84)	13 (100)
*Sentiment*			
Positive	5 (22)	23 (54)	8 (62)
Negative	5 (22)	2 (5)	1 (8)
Neutral	13 (56)	18 (41)	4 (30)
*Themes*			
Person’s name	10 (44)	1 (2)	1 (8)
Funny/playful	3 (13)	0 (0)	0 (0)
About dental health	3 (13)	2 (5)	0 (0)
About retainers	1 (4)	1 (2)	2 (15)
About campaign	2 (9)	1 (2)	3 (23)
Positive support	3 (13)	18 (42)	6 (46)
Negative	1 (4)	0 (0)	0 (0)
Miscellaneous	0 (0)	20 (47)	1 (8)

Values are given as n (%).

## Discussion

This cross-sectional study has investigated exposure of the UK #HoldthatSmile national orthodontic retention awareness campaign through content analysis of posts identified on Facebook, Instagram and Twitter over a 12-month period immediately following campaign launch. A total of 205 separate relevant posts were identified through these platforms with a calculated audience reach of over 108,000 individuals. Despite having the lowest number of posts and population reach, Instagram was associated with the highest level of audience engagement. Most users were either individual dental surgeries or professional dental bodies. Content analysis of posts revealed that the majority expressed overall positive or neutral sentiments. Predominant themes within the comments were user identity as individuals bringing #HoldthatSmile to the attention of their peer-groups, and positive support.

Studies investigating the use of SMPs for health campaigns are an emerging area of research and, to date, most have focussed on those associated with the medical specialties (Lenoir et al., 2017; Thackeray et al., 2013). This is the first analysis of social media activity following a national awareness campaign in orthodontics. The number of posts relating to this campaign was relatively low given the 12-month period of data collection, ranking in the hundreds rather than hundreds of thousands seen for other healthcare campaigns (Thackeray et al., 2013); however, public health bodies commonly have problems engaging the public in health campaigns and delivery of health information overall (Klassen et al., 2018; Neiger et al., 2013). In addition, the analysis may have underestimated public engagement, as the initial campaign was targeted at the profession and, anecdotally, it seems that some used it in waiting rooms for patients. So potentially, the SMPs were used to highlight awareness among professionals who then passed information on to patients through videos. Interestingly, the calculated audience reach from these posts extended to over 100,000 individuals, which demonstrates the power of SMPs in potentially disseminating information. Among the posts identified in response to this campaign, there was a much higher reach potential associated with Facebook compared to Instagram and Twitter. This is consistent with data from similar campaigns (George et al., 2018) and is almost certainly related to the fact that Facebook remains the top global SMP with over 2 billion active users (Statista, 2020).

However, the lack of engagement by Facebook users does suggest that a lot of them are passive, which potentially compromises the platform in its ability to promote behavioural change. Instagram was the most effective SMP at engaging users for #HoldthatSmile, which correlates with a recent study investigating a social media campaign for user uptake relating to an Australian health programme 10,000 Steps (Rayward et al., 2019). Interestingly, this project also demonstrated the significant effect of paid advertisement in the promotion of an awareness campaign. #HoldthatSmile did not use paid advertisements, relying on organically generated posts, which might have further contributed to the relatively low volume of posts associated with this campaign.

The majority of posts about #HoldthatSmile were from dental surgeries or professional organisations within dentistry. Given that the campaign was launched by a national body that promotes the practice of orthodontics, this is perhaps not surprising. Indeed, the campaign provoked an opinion piece in the *British Dental Journal*, highlighting aspects of the campaign from the point of view of a general dental practitioner (Crory, 2018) with an associated response from BOS representation (Littlewood and BOS, 2018). However, the low number of users from the general public suggests that this target population was either not reached or not interested. Patients and parents are the groups who would potentially benefit most from the campaign messages and to whom these messages were ultimately directed. A further aim of the campaign was to provide a reliable source of information on orthodontic retention for the public. Data relating to this aspect may be better represented by analysis of visits to the BOS website, views of the three retention videos and through traffic to the retention resources from links via SMP. These are areas that could be analysed in a further investigation but were beyond the scope of this study. Furthermore, the YouTube informative videos were available for dental practices to download and play in their waiting rooms to educate the public about orthodontic retention, another area that might benefit from feedback. There were many facets to the campaign and this study has only investigated the social media aspect.

The general sentiment of the comments on SMPs showed the campaign to have been positively received in general, more on Instagram and Twitter than Facebook. Indeed, the qualitative interpretation of comments showed there to be overall positive support for the campaign on Instagram. There were tendencies for users to continue the spread of information for the campaign by targeting further specific users through the inclusion of their names. There were also questions raised within some of the comments about dental health and retainers, demonstrating further scope for educating the public.

There is no doubt that a campaign of this type is to be commended, offering a simple set of messages on an important subject for orthodontic patients. It is important for BOS members to engage fully with these types of campaigns, actively promoting them in their online messaging. Current information on the Internet relating to retainer wear is easily accessible, but not always accurate and reliable, with only a minority of websites advocating indefinite wear of removable and fixed retainers (Dogramaci and Rossi-Fedele, 2016). Similar findings have also been reported from studies investigating information quality available on the Internet for other aspects of orthodontic treatment, including extractions (Patel and Cobourne, 2011), pain (Livas et al., 2013) and adult treatment (McMorrow and Millett, 2016). The lack of trustworthy online orthodontic resources means there is a professional duty, to ensure there is reliable, evidence-based information is available in which patients can be signposted to and by platforms most accessible and utilised by the public. This, in turn, can only be advantageous for clinicians in supporting dialogues about treatment and building the patient–clinician rapport. A key issue for SMS campaigns of this type is engaging with the public as well as professionals. The public reach here was relatively small by social media standards although the there is no real data relating to the metrics of successful online campaigns in oral health. Another issue is encouraging wider engagement while maintaining a professional and informative approach to the campaign. A paid campaign might have increased numbers but there are multiple issues in orchestrating this type of project.

### Limitations

SMPs by their very nature are dynamic and accessible by users 24 hours a day. Consequently, content and follower counts are updated and changed frequently. Data collection for each platform would ideally be carried out simultaneously to ensure optimum accuracy. However, this is only achievable using automated specialised programmes not available in this study. Data collection and interpretation were therefore limited to manual practicable limits. There are also an almost limitless number of metrics that can be utilised to analyse the performance of SMP, particularly in association with marketing campaigns. This study confined data analysis to established parameters used within all SMPs, in order to make the data comparable and workable. Moreover, this area is by its very nature fluent, and it is likely that a different response would be elicited by a similar campaign carried out now.

## Conclusion

The first 12 months of the BOS #HoldthatSmile campaign generated a relatively low number of posts on Twitter, Facebook and Instagram. However, these were associated with an audience reach in excess of 100,000 individuals with the majority conveying positive or neutral messages. The study found a variance in performance of the individual SMP, suggesting a difference in their use by the public which could be utilised by the professions: Twitter for dissemination of information, Facebook for exposure and Instagram for engagement.
